# From 4D Medical Images (CT, MRI, and Ultrasound) to 4D Structured Mesh Models of the Left Ventricular Endocardium for Patient-Specific Simulations

**DOI:** 10.1155/2018/7030718

**Published:** 2018-01-08

**Authors:** Federico Canè, Benedict Verhegghe, Matthieu De Beule, Philippe B. Bertrand, Rob J. Van der Geest, Patrick Segers, Gianluca De Santis

**Affiliations:** ^1^IBiTech-bioMMeda, Department of Electronics and Information Systems, Ghent University, Ghent, Belgium; ^2^FEops NV, Ghent, Belgium; ^3^Department of Cardiology, Ziekenhuis Oost-Limburg, Schiepse Bos 6, 3600 Genk, Belgium; ^4^Division of Image Processing, Department of Radiology, Leiden University Medical Center, Leiden, Netherlands

## Abstract

With cardiovascular disease (CVD) remaining the primary cause of death worldwide, early detection of CVDs becomes essential. The intracardiac flow is an important component of ventricular function, motion kinetics, wash-out of ventricular chambers, and ventricular energetics. Coupling between Computational Fluid Dynamics (CFD) simulations and medical images can play a fundamental role in terms of patient-specific diagnostic tools. From a technical perspective, CFD simulations with moving boundaries could easily lead to negative volumes errors and the sudden failure of the simulation. The generation of high-quality 4D meshes (3D in space + time) with 1-to-1 vertex becomes essential to perform a CFD simulation with moving boundaries. In this context, we developed a semiautomatic morphing tool able to create 4D high-quality structured meshes starting from a segmented 4D dataset. To prove the versatility and efficiency, the method was tested on three different 4D datasets (Ultrasound, MRI, and CT) by evaluating the quality and accuracy of the resulting 4D meshes. Furthermore, an estimation of some physiological quantities is accomplished for the 4D CT reconstruction. Future research will aim at extending the region of interest, further automation of the meshing algorithm, and generating structured hexahedral mesh models both for the blood and myocardial volume.

## 1. Introduction

Cardiovascular disease (CVD) is the leading cause of death, with a worldwide mortality of 31% [1] and even 40% within the European borders [2]. In absolute terms, 17.5 million people die each year in the world from CVDs. Not surprisingly, numerous studies focused on a better understanding of CVDs including hypertension, atherosclerosis, Coronary Heart Disease (CHD), stroke, Congestive Heart Failure (CHF), and congenital and rheumatic heart disease. The clinical diagnosis of CVDs most often relies on different imaging modalities such as angiography, MRI, Ultrasound (US), and CT with the choice depending on the pathology under investigation.

Thanks to the massive development of computational resources, in the last 20 years there has been a growing interest in computational modeling such as Computational Fluid Dynamics (CFD) and Finite Element (FE) and Fluid-Structure Interaction (FSI) analysis. Nowadays, applied biomechanical research has moved into the direction of medical and patient-specific applications; thus the coupling between clinical images and computational modeling can play a fundamental role in this regard. Several examples of commercially available simulation-based services can be mentioned: Medis [3] and HeartFlow [4] use CFD as diagnostic tool for Fractional Flow Reserve (FRR) by means of angio-based and CT-based images, respectively, while from a treatment perspective FluidDA [5] and FEops [6, 7] developed commercial tools to support clinicians in respiratory diseases cases and in TAVI applications. Lastly, SIMULIA created an integrative predictive biophysical model of the human heart to extract clinical parameters and guide device design and treatment planning in cardiac diseases [8].

While vascular pathologies can—depending on the specific application—be addressed using models not accounting for the dynamic expansion and recoil of the wall, modeling of the flow or the mechanics of the heart chambers cannot disregard the large motion of cardiovascular structures. Indeed, the combination and integration of computational modeling and clinical images become even more essential. Among the 4 chambers, the left ventricle (LV) is the pump responsible for the systemic circulation and a better knowledge of its fluid dynamics can help to understand pathologies such as hypertrophy, valvular leakages, cardiomyopathy, and correct phenotyping of heart failure. In this context, the general final target of this work is to develop a pipeline that allows setting up patient-specific CFD models of the LV that are able to quantify the swirling flow inside it, which could help the clinicians in diagnosing, phenotyping, and finding the best therapy.

The CFD modeling of a heart chamber, such as the LV, faces important technical obstacles such as accounting for endocardial motion and valves kinematics. The rapid and large motion and deformation of these features can easily lead to very distorted elements in the mesh that is used for the CFD calculation and affect its accuracy or in the worst case scenario lead to negative volume meshes and, thus, failure of the simulation.

Despite a lot of works focusing on 4D (3D space + time) LV segmentation (mainly based on deformable models and Statistical Shape Modeling (SSM)) to the best of our knowledge there is still a lack of methodologies for high-quality structured meshing of a 4D dataset for subsequent CFD analysis.

Among the deformable models, one can cite the studies by Wang et al. [9] and Gao et al. [10] who reconstructed the 4D LV, the former by meshless point-clouds and the latter including anatomical details such as the papillary muscles (PMs) and the trabeculae. Bavo et al. [11] reproduced the LV with a semiautomated image segmentation and the mitral valve (MV) [12] with a combination of multiatlas joint label fusion and deformable models. Škrinjar and Bistoquet [13] built meshes from segmented 3D MR images mapping a premeshed sphere to the target surface of the segmented object with the mapping operation defined by the gradient field of the solution of the Laplace equation between the sphere and the surface of the object.

Among the SSM approaches the studies of Sun et al. [14] and Zhu et al. [15] used the shape knowledge combined with dynamic information to account for cardiac shape variability, while Besbes et al. [16] used a control point representation of the LV prior to deforming other images.

When using deformable meshes derived from 4D data sources, related key issues are sensitiveness to the initialization process with manual interaction required for the first step, poor convergence for noisy images, and large errors associated with large deformations between neighboring frames.

As far as we know, only two studies (Schenkel et al. [17] and Doenst et al. [18]) based their CFD analysis in the LV on structured hexahedral meshes. These were semiautomatically created by projecting the vertexes of a tube-like base grid onto the segmented surface. No detailed information about the followed strategy was reported in the paper. In Schenkel et al. [17], the hexahedral mesh is generated following the same strategy but for the whole heart. Nonetheless, the authors report that the quality of the mesh could not be preserved with meshes exceeding 400,000 elements. Lim et al. [19] realized a 4D patient-specific mesh of the right heart from segmented MRI contour lines with 1-to-1 vertex correspondence in 2013 and, in the following year, the method was extended to both the left and right heart [20]. In both the studies, the delineation of the contour lines had to be drawn across all slices through the cardiac cycle, resulting in a time-consuming operation. Furthermore, only triangular surface meshes are presented in these studies, while in the CFD community the use of a hexahedral or hybrid meshes with a prism layer is preferred for a more accurate Wall Shear Stress (WSS) evaluation [21].

Consequently, we believe that there is still a lack of practically feasible methodology to derive high-quality structured meshes from 4D clinical cardiac imaging dataset with 1-to-1 vertex correspondence and this study attempts to fill this gap. The nodal correspondence between the 4D meshes is essential in a CFD set-up with moving boundaries to assign the endocardial wall displacement as the displacement of each node that composes the mesh and avoids problems of mesh quality that could be induced by spatial interpolation schemes. Furthermore, as a result of temporal interpolation, the nodal correspondence of the 4D high-quality meshes allows us to generate high-quality intermediate meshes and overcome, in this way, the low temporal resolution of 4D clinical imaging datasets for a CFD software environment.

In the following sections, the steps of the newly proposed mesh generation methodology are described in detail, starting from the general strategy [Section 2.3], as well as its applications on the different parts that compose our ventricle mesh [Sections 2.4, 2.5, and 2.6]. In order to demonstrate the versatility and efficiency, it is tested with the three most-used 4D cardiac imaging modalities (CT, US, and MRI). Lastly, the quality and the accuracy of the different meshes are analyzed.

## 2. Methods

**Figure 1:**
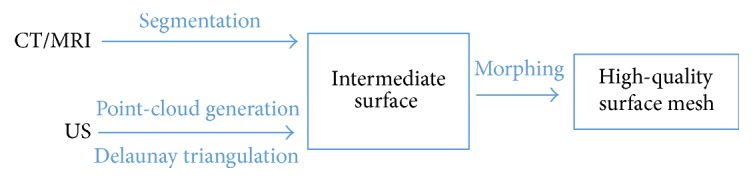
The workflow used for the three different 4D datasets is reported: CT and MRI datasets are segmented by using the Materialise Mimics® CT Heart tool, whereas the US images are segmented via an in-house code developed in PyFormex, a python-based software environment. The morphing is performed with algorithms that rely on isoparametric transformations.

The high-quality mesh tool will be tested on three different types of 4D imaging datasets (US, MRI, and CT) coming from three different patients in order to prove the versatility and the applicability of the method. The segmentation procedure is differently managed depending on the availability of the segmentation software (Figure 1), while the high-quality meshing scripts have been developed in PyFormex, a python-based open-source software (http://www.nongnu.org/pyformex/).

### 2.1. Datasets

All images were acquired as part of standard clinical care in 3 different hospitals. Data were anonymized and all subjects gave informed consent for the use of their data for this study. The 4D MRI dataset was acquired along the short axis of the ventricle with an initial slice thickness of 10 mm. Due to the high slice thickness the data were interpolated by means of trilinear interpolation resulting in a slice thickness of 2 mm, whereas no interpolation was used for the US and CT datasets. The voxel dimensions are 1 × 1 × 1 mm, 1.641 × 1.641 × 2 mm, and 0.352 × 0.352 × 0.75 mm, respectively, for 4D US, 4D MRI, and 4D CT. For each imaging modality, data spanning one cardiac cycle were available with data at 23 time instants for 4D US, 30 for 4D MRI, and 15 for the 4D CT.

### 2.2. Segmentation

In the case of the 4D CT dataset the segmentation was performed straightway using the Materialise Mimics CT Heart tool for heart chamber segmentation for each of the 15 time frames of the cardiac cycle. Also each frame of the 4D MRI dataset (30 time frames) was initially segmented by means of the Materialise Mimics CT Heart tool but due to the low through-plane spatial resolution manual corrections were needed. For the CT and MRI datasets also other open-source software (such as 3D Slicer) could have been used, but to speed up the process these other ways were preferred.

Conversely, as far as we know there are no available open-source software able to visualize and segment 4D Ultrasound datasets. For this reason, we developed our own algorithm compiled in PyFormex, a python-based in-house software environment, to manually segment this dataset.

**Figure 2:**
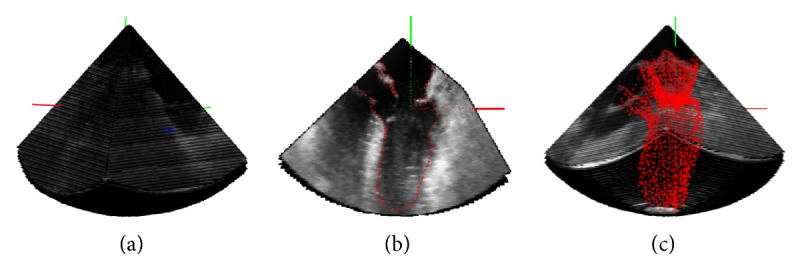
Steps of the algorithm to reconstruct the 4D point-clouds from the 4D US dataset: (a) definition of the rotating axis; (b) placement of the points in a view of the US cone; (c) resulting point-cloud distribution of the LV.

The workflow of the algorithm can be described in 5 steps:

(1) Load the 4D US dataset and define the axis of the dataset along which the camera is rotated (Figure 2(a)).

(2) Manually place points in the view to delineate the LV endocardium (Figure 2(b)).

(3) Rotate every 5 or 10 degrees around the rotating axis and repeat Step  (2) until the complete volume of the chamber is covered (Figure 2(c)).

(4) Save the point-cloud of the LV for each time-frame (time-frame can be manually chosen by rolling a scroll-bar).

(5) As outcome of this process, *N* left ventricle point-clouds are obtained, where *N* is equal to the number of time points where data is available and the surfaces are obtained by means of Delaunay triangulation.

### 2.3. High-Quality Mesh Generation: Morphing Tool

**Figure 3:**
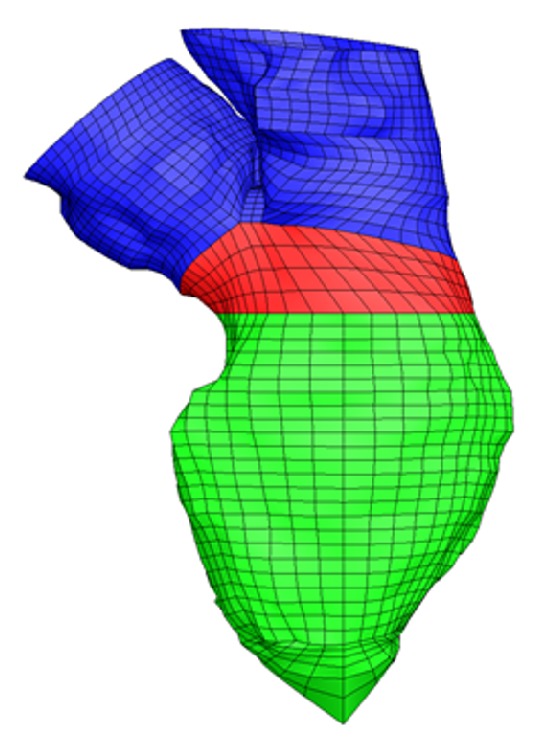
LV divided into three parts: LV sac (in green), Y-junction (in blue), and connection between the two (in red) [4D CT dataset].

In this core section, the strategy behind the generation of the high-quality structured mesh is described and implemented as semiautomatic process to the different parts that compound the LV (Figure 3): LV sac, Y-junction, and connection between the LV sac and Y-junction. The tool is described for the CT dataset, but it is generic and has been used for the processing of all datasets in this paper. We choose to detail the method using the CT dataset as this is the most complex dataset that includes the LV as well as the left atrium, aorta, and the valvular planes. As it is impossible to mesh these structures as a whole, 3 subdomains are created ((i) the ventricular sac, (ii) the atrium and aorta, and (iii) the zone connecting both). After discerning the general strategy of the high-quality mesh generation, it will be explained how these 3 subdomains can be meshed using a morphing toolkit specifically designed for these 3 anatomical regions.

**Figure 4:**
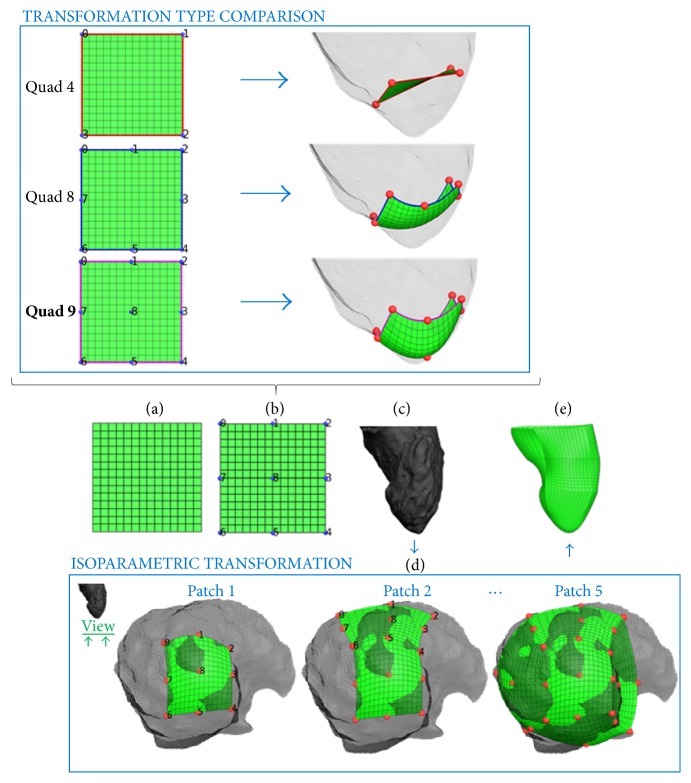
Example of the strategy used to reconstruct the target geometry by means of isoparametric transformations: (a) definition of the patch in the *x*-*y* domain used to cover the target geometry; (b) definition of the initial control points (CPs) of the isoparametric transformation; (c) initial geometry to be mapped; (d) placement of the final CPs onto the target geometry and sequential application of the isoparametric transformations; (e) final mapped geometry. The top part of the figure shows how the same patch is differently deformed using different isoparametric transformations (quad 4, quad 8, and quad 9).

The general strategy is to cover the segmented surface of the 4D dataset, which typically has a nonadequate quality of the surface mesh, with a combination of patches that have the desired mesh topology by means of isoparametric transformations. The application of an isoparametric transformation requires the definition of the following inputs:

(i) The patch: this is the object (2D or 3D) to which to apply the isoparametric transformation. In our case, the patch is a structured quadratic surface mesh defined in the *x*-*y* plane (2D) (Figure 4(a)), but it could be of any shape (a triangular mesh, a star, an asterisk, etc.).

(ii) The initial control points (CPs): these are defined on the patch to be transformed (Figure 4(b), in blue). Their number (equal to the number of the final CPs) depends on the type of transformation.

(iii) The final CPs: these are the points, numbered identical as the initial CPs, onto which the initial CPs will be transformed (Figure 4(d), in red). In other words, their location will define the shape of the resulting patch.

(iv) The type of transformation: this sets the type of relation between the initial and final CPs. For example, a quadratic transformation can have 4 CPs (in the corners of the patch), 8 CPs (in the corners and in the middle of the edges), or 9 CPs (with the extra CP located in the center of the face) (Figure 4). In the quad 4 transformation the edges of the transformed object follow a linear equation and in the quad 8 and quad 9 a quadratic one [21]. In our case, a quad 9 transformation is used because it allows a more accurate adhesion to the irregular structures that compose the LV endocardium (as visible in the top panel of Figure 4). To be more specific, in case of quad 9 isoparametric transformation, the transformation of a point (*x*, *y*, *z* = 0) to a new point (*x*_1_, *y*_1_, *z*_1_) is described by the following 2nd-order Lagrangian interpolation polynomial in *x*, *y*:

(1) x1=a0+a1·x+a2·y+a3·x2+a4·y2+a5·x·y+a6·x2·y+a7·x·y2+a8·x2·y2y1=b0+b1·x+b2·y+b3·x2+b4·y2+b5·x·y+b6·x2·y+b7·x·y2+b8·x2·y2z1=c0+c1·x+c2·y+c3·x2+c4·y2+c5·x·y+c6·x2·y+c7·x·y2+c8·x2·y2,

where the coefficients (*a*_*n*_, *b*_*n*_, *c*_*n*_) are determined by solving the system of linear equations expressing the conditions that the initial control points should map exactly on the final ones. For example, assuming that the first initial control point is (1, 2, 0) and the final one is (4, 6, 7) the corresponding set of equations becomes:

(2) 4=a0+a1·1+a2·2+a3·12+⋯+a8·12·226=b0+b1·1+b2·2+b3·12+⋯+b8·12·227=c0+c1·1+c2·2+c3·12+⋯+c8·12·22.

By enforcing the conditions for interpolation for each of the control points, the coefficients can be determined and the above equations with the known coefficients are used to compute the new position of each node in the patch. Note that the quadratic edges of the final mesh would introduce a discontinuity in the tangent where the border nodes connect two consecutive patches. This problem can be tackled by using an isoparametric transformation with cubic edges (quad 16 transformation) or, as we did, by increasing the density of the patches until this effect becomes negligible.

As outcome of the isoparametric transformation, the patch is moved from the initial CPs to the final ones by following the defined transformation. In our application the strategy is to place the final CPs on the LV endocardium for every segmented surface of the 4D dataset and to apply the isoparametric transformation sequentially until the surface is completely covered by the defined patch (Figure 4(e)).

In this way the initial patch is deformed accordingly to the shape of the geometry that the user wants to reproduce. The effects of this method can be qualitatively evaluated by comparing Figures 4(c) and 4(e), where the former figure represents the initial segmented geometry and the latter the high-quality mesh of the reproduced object. In the case that the user wants to include finer details of the initial geometry, more CPs have to be used. There is not any restrain on which geometry of the 4D dataset to start with.

The accuracy of this method is proportional to the number of CPs used, because the new mesh is anchored to the old one by means of the CPs, which are exactly coincident with the surface, while the intermediate nodes are not necessarily exactly on the surface.

Dedicated algorithms have been developed to semiautomatically project the final CPs onto all the configurations of the 4D dataset in order to avoid a tedious and a time-consuming manual positioning of the final CPs in each of the LV target configurations that form the 4D datasets.

Note that a simple radial projection of the CPs from a scaled down model on the whole geometry would have resulted in an inaccurate reproduction in particular around the Y-junction area (where the LV is connected to the LA and aorta). For this reason, the mesh of the LV is built as sum of three different parts (the LV sac, the Y-junction, and the connection between these two parts) and disclosed separately in the following Sections 2.3.1, 2.3.2, and 2.3.3.

The low spatial resolution of the 4D MRI and 4D US (if compared to the 4D CT) could result in high uncertainty in correspondence of the Y-junction, where finer details would be needed. Consequently, only the LV sac is built for the 4D MRI and 4D US datasets, while the complete reconstruction is performed for the 4D CT dataset.

#### 2.3.1. Application of the Morphing Tool Application on the LV Sac

**Figure 5:**
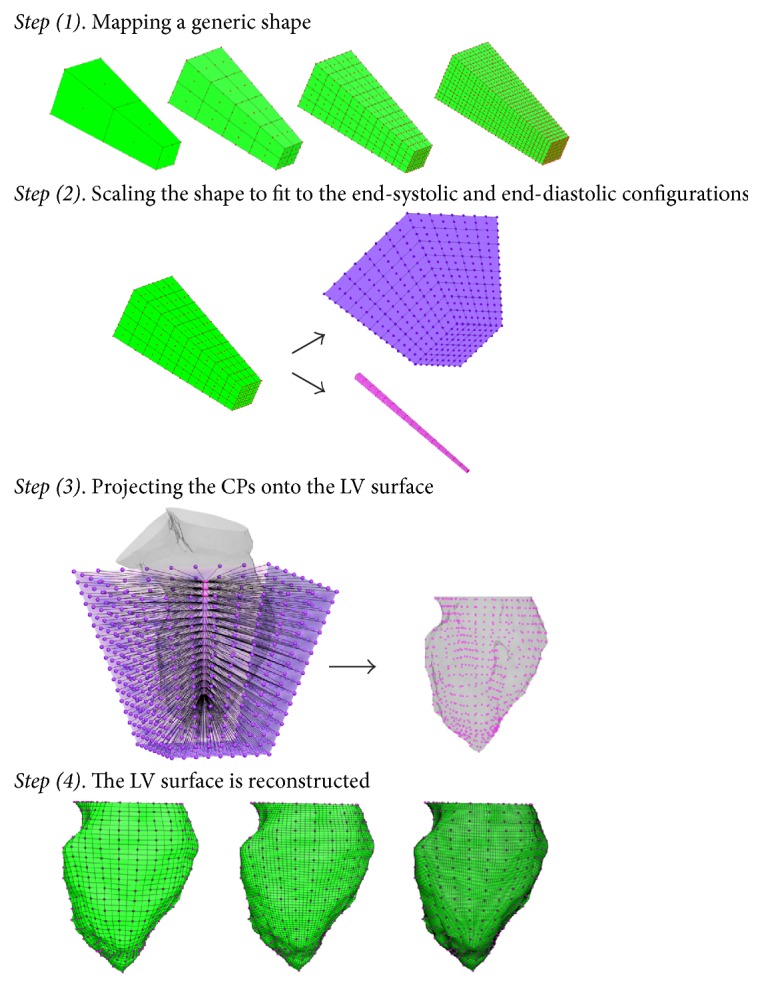
Strategy used to build a high-quality mesh of the LV chamber: (1) a truncated pyramid is mapped with the desired mesh topology (resp., 9, 36, 144, and 420 patches from left to right); (2) the mapped truncated pyramid is scaled up and down to fit completely inside and outside the LV; the CPs of the two scaled truncated pyramids define the projection trajectories to be followed to locate the CPs onto the target LV; (3) the CPs are projected onto the LV surface; (4) the LV surface is reconstructed by using the projected CPs and the connectivity of the truncated pyramid. The user can directly choose the number of nodes by varying the number of subdivisions of the patches.

In this section, the strategy used to project the final CPs of the isoparametric transformation on the LV sac configurations of the 4D dataset is reported. The structured mesh of the LV sac is built up to a predefined percentage of the total vertical length of the LV, from the apex to the mitral valve orifice centroid. The application of the morphing tool in the LV sac can be summarized as follows:

(1) Build a simplified generic shape (Figure 5(1)) (whether half cylinder or truncated pyramid) to which the manual morphing tool is applied with the desired number of patches and CPs. In Figure 5(1), the simplified generic shapes used with 9, 36, 144, and 420 patches (from left to right) are reported.

(2) Scale up and down the mapped simplified generic shape in order to fit outside and inside the LV (Figure 5(2)). The CPs of the two scaled models define the projection trajectories of the CPs onto the target surface.

(3) The CPs are projected onto the target surface by following the defined projection trajectories (Figure 5(3)).

(4) The LV sac is mapped with the same patches of the simplified generic shape. The user has a direct control on the number of nodes by changing the number of subdivisions inside each patch (Figure 5(4)).

The morphing tool application in the LV sac is performed sequentially to all the configurations of the 4D dataset by using a loop-structure. The scale factors of Step  (2) are chosen such that the two scaled models fit completely inside and outside all the LV configurations throughout the cardiac cycle. Therefore, the scale down and scale up factor are tuned manually at this stage with respect to the end-systolic (such that one scaled model fits inside) and end-diastolic configurations (the second scaled model should encompass this configuration).

Once built and mapped, one simplified generic shape can be used to map an infinite number of datasets. Therefore, Step  (1) has to be repeated only if the user wants to change the number of CPs.

#### 2.3.2. Application of the Morphing Tool to the Y-Junction (Aorta and Left Atrium)

As already mentioned this step was accomplished only for the 4D CT dataset. Anatomically the left atrium and the aorta root are very closely located resulting in the empty space between the two parts to be very narrow. For this reason, the previous approach of deforming the initial mapped shape (truncated pyramid) into the 4D segmented shapes was feasible only for the LV sac reconstruction of the different 4D imaging datasets. However, the same approach was found impracticable for the upper part of the left heart (denoted as Y-junction), because in this region the nodes of the LA and of the aorta are separated by a really thin empty space which would result intersected after the deformation to the new temporal configuration.

Therefore, this anatomical part is treated as a vessel bifurcation and mapped by means of the following these steps.

**Figure 6:**
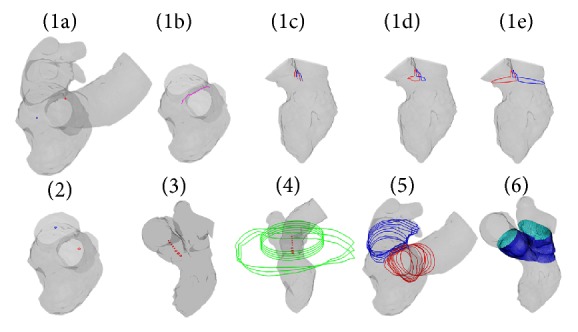
Strategy followed to create high-quality mesh of the Y-junction: (1) generation of two biplanar meshes; (2) generation of two circular meshes infinitesimally small located in the centroid of the valvular planes; (3) translation of the circular mesh up to the desired position; generation of intermediate Polylines infinitesimally small (in red) as linear interpolation between a concentric scaled down biplanar meshes and the translated circular meshes; (4) the intermediate small Polylines are concentrically scaled up (in green); (5) the Polylines of the bifurcation are created as linear intersection between the small and big versions of the Polylines which lie on the target surface; (6) the coordinates of the Polylines are used as final CPs for the isoparametric transformations.

1. Two biplanar meshes are located in the bifurcation center (Figure 6(1e)) by following this procedure:
  (1a) Two points are manually picked in the part of the surface that separates the conduits (Figure 6(1a)).
  (1b) A Polyline is delineated as a result of the intersection on the surface between the Polyline passing through the two points and a copy of it translated in the vertical direction (Figure 6(1b)).
  (1c) The resulting Polyline is translated towards the two directions of the valve centroids (Figure 6(1c)).
  (1d) The two translated Polylines are rotated to obtain a double upside-down Y-mesh in correspondence to the bifurcation center (Figure 6(1d)).
  (1e) The nodes of the horizontal semicircle are radially projected onto the target surface (Figure 6(1e)).
2. An infinitesimally small circular shape mesh-line, that shares the same topology of the biplanar mesh, is located in the centroid of the two valvular planes (Figure 6(2)).
3. The circular meshes are translated up to the desired vertical length; N small intermediate Polylines are created as a result of linear interpolation between a concentric scaled down biplanar mesh and the circular shape mesh-line (Figure 6(3)).
4. N large intermediate Polylines are created by scaling up concentrically the N small intermediate Polylines (Figure 6(4)).
5. Polylines of the conduits are created as a result of the linear intersection between the intermediate small and big Polylines on the target surface (Figure 6(5)).
6. The coordinates of the Polylines are used as target CPs of the isoparametric transformations (Figure 6(6)).

As before, the morphing tool is applied sequentially to all the configurations of the 4D dataset by using a loop-structure. Only Step  (1a) requires manual interaction of the user in order to pick the two points in the first configuration of the datasets. The remaining steps are performed automatically by the algorithm. In the current state, the main limitation of the Y-junction reconstruction is that the LA appendage cannot be reproduced accurately and for this reason it was not included during the segmentation process.

#### 2.3.3. Connection of the LV Sac and the Y-Junction

**Figure 7:**
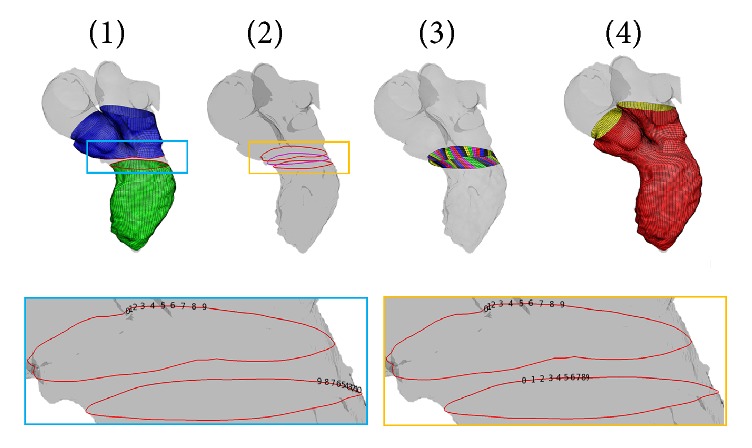
Generation of the connection between LV sac and the Y-junction: (1) renumbering of the border nodes; (2) generation of an intermediate Polyline; the nodes of the three Polylines will be the final CPs of the isoparametric transformations; (3) application of the isoparametric transformation; (4) final reconstruction of the total LV.

The upper LV sac border and the lower border of the Y-junction of each mesh are built such that they share the same number of nodes but not the same numbered order. The final step is then to connect the two parts following this strategy:

(1) Reordering the nodes of the two borders (ventricle-side and bifurcation-side) (Figure 7(1)). This intermediate step is needed because the generated lower border of the bifurcation and the upper border of the LV sac do not share the same numbering of nodes.

(2) Generation of an intermediate Polyline that lies on the target surface (Figure 7(2)).

(3) The coordinates of the two borders and the intermediate Polyline are used as final CPs for the isoparametric transformations (Figure 7(3)).

The morphing tool application in the LV sac, Y-junction, and the connection between these two parts is performed sequentially for every LV configuration of the 4D dataset by means of a loop-structure.

### 2.4. Temporal Interpolation

**Figure 8:**
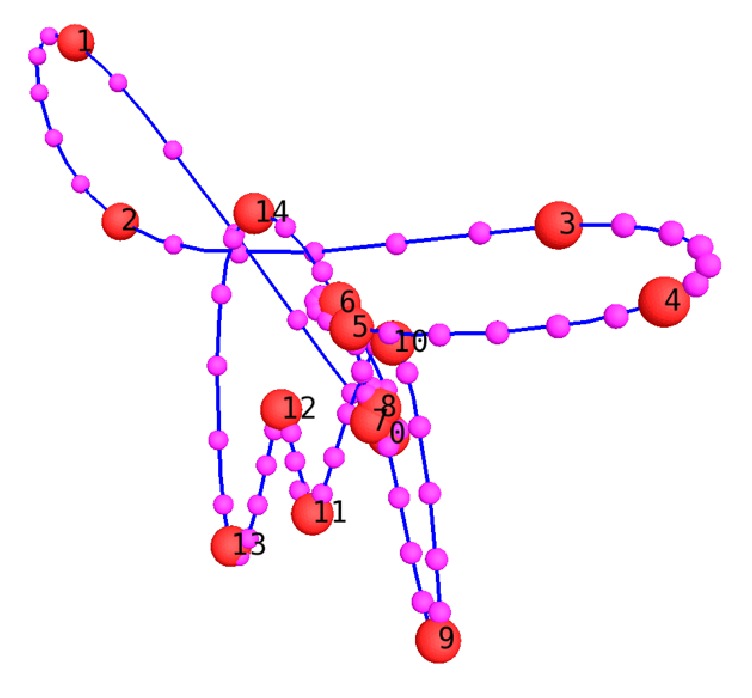
Temporal interpolation for the first node of the 4D reconstructed meshes during a cardiac cycle, represented as trajectory (in blue) between the positions derived from the 4D CT reconstruction with 336 CPs (in red, numbered from 0 to 14). The resulting intermediate positions of each node (in magenta, for the first node) are connected using the initial mesh connectivity to get the intermediate meshes.

The temporal interpolation of the newly created 4D structured meshes is needed in order to match the temporal resolution requirements of a CFD simulation (Figure 8). In this way, the set-up of the CFD model with moving boundaries is independent of the time instants available in the 4D imaging dataset.

The related code (developed as well in PyFormex) takes the 4D newly created meshes as input and interpolates the position of each node of the mesh with a cubic spline (Bezier or natural) that can be chosen by the user. By knowing the cardiac cycle duration and the time step size required in the CFD simulation, the user can choose the number of the intermediate configurations to be created.

### 2.5. Quality and Accuracy Assessment of Generated Meshes

In order to validate the described method both the quality of the meshes and the accuracy of the meshed geometry reconstructions, compared to the starting dataset, are investigated.

As quality criterion the equiangle skewness (Qeas) is chosen. This parameter, often used as quality index for CFD meshes, represents a normalized measure of skewness, ranging from 0 (for an optimal equilateral cell) to 1 (for a completely degenerated cell). The Qeas depends on the angle formed between adjacent edges of each cell in the mesh:

(3) $$\begin{array}{c} Qeas=\max\left(\;\frac{\vartheta \max\;-\vartheta e}{180-\vartheta e},\frac{\vartheta e-\vartheta \min\;}{\vartheta e}\;\right), \end{array}$$

where *ϑ*max⁡  and *ϑ*min⁡  are the largest and smallest angles in the cell and *ϑe* is the angle of the equiangular cell, which is 90° for the quadrilateral faces. As is usually done in the CFD meshing community, the maximum and average threshold values of the equiangle skewness (from now on referred to simply as skewness) are set, respectively, at 0.9 and 0.25 [22–24].

Regarding the accuracy, the distances of the points in the mapped mesh from the segmented surface are reported for all the 4D reconstructed datasets. The distance for each node of the mapped mesh from the segmented surface has been classified into 6 fixed different intervals from 1/1000 of mm up to the maximum computed value. We also calculated the volume percentage difference with respect to the initial segmented geometries. This was done only for the 4D CT dataset because only in this case the LV was completely reconstructed.

### 2.6. Algorithm Speed Assessment

The analysis was performed on a PC with a processor Intel® Core™ I7-4810MQ @2.80 GHz with 16.0 GB RAM. The total time of the algorithm is directly reported by PyFormex while the time related to reading operations has been evaluated using the timeit function available in the timeit python library.

## 3. Results

For the 4D CT dataset 336, 968, and 2165 CPs were chosen to reconstruct the complete LV, while for the 4D US and 4D MRI datasets the LV sac reconstructions were performed with 41, 153, and 593 CPs. This different choice is due to the fact that for the 4D CT dataset we reproduced the whole LV geometry and (at least partially) finer details of the endocardial surface such as the trabeculae.

For clarity the analysis of the parameters is conducted on averaged values over a dataset (all time steps), except when reporting the maximum and minimum values of a parameter. Unless explicitly stated, conclusions drawn are applicable to each mesh of the 4D dataset. The skewness distribution is reported for the reconstructed LV mesh of the 4D data that has the maximum skewness value.

### 3.1. CT Dataset

**Figure 9:**
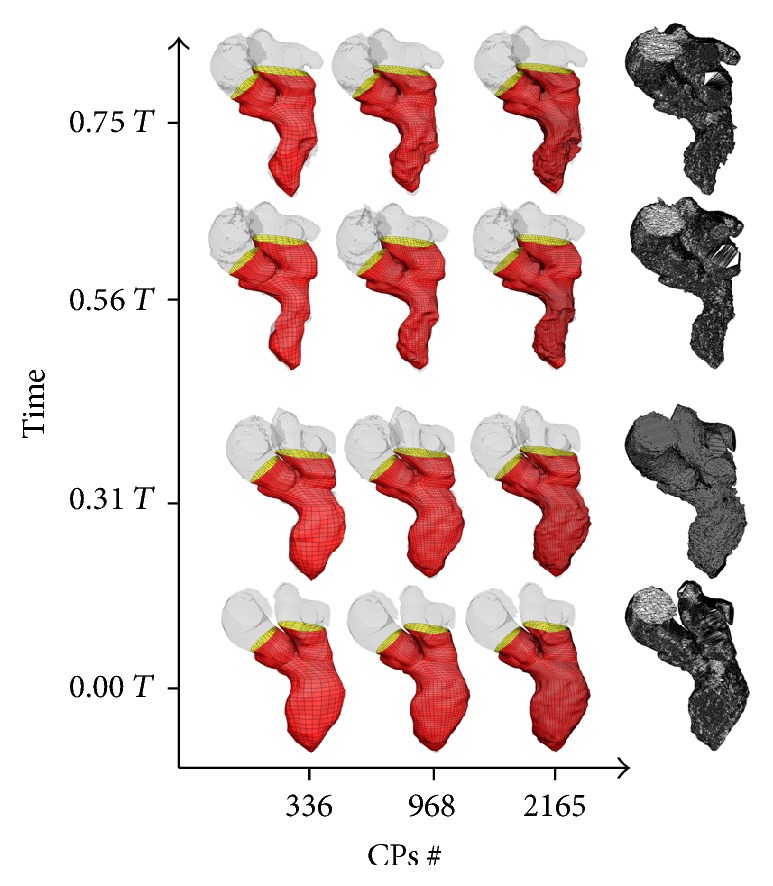
LV sac reconstructions for the 4D CT dataset with 336 (left), 968 (middle), and 2165 (right) at the end-diastolic configuration (1st row down), end-systolic configuration (3rd row down) CPs, and two intermediate configurations (2nd and 4th rows). On the right side of the graph are the corresponding segmented meshes. *T* represents the adimensional period of the cardiac cycle, where *T* = 0 indicates the time corresponding to the end-diastolic configuration.

For the 4D CT dataset the LV sac was reconstructed with 153, 593, and 1705 CPs. In the first case the reconstruction resulted in smooth LV endocardial surfaces, while in the other cases the finer control given by the increased number of CPs was allowed to catch, at least partially, the trabeculae and the papillary muscle. The Y-junction was reconstructed, respectively, with 183, 375, and 460 CPs. Therefore 3 different reconstructions of the total geometry (which includes the LV, the left atrium, aorta, and the valvular planes) were performed with 336, 968, and 2165 CPs (Figure 9). In addition to the accuracy and quality evaluations (for the 4D CT dataset) an estimation of some useful physiological quantities is disclosed.

#### 3.1.1. Accuracy

**Figure 10:**
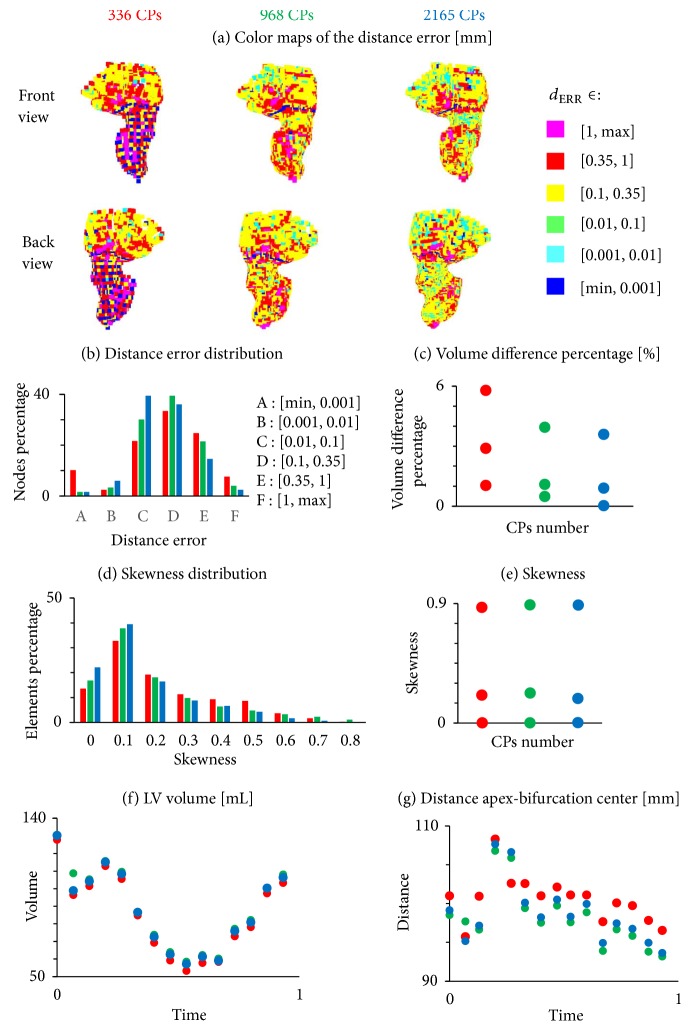
Accuracy of the generated 4D CT meshes from the original 4D dataset (a), (b), (c); quality of the newly created 4D meshes (d), (e); estimation of physiological parameters (f), (g). In Figure 9(a), the color maps of the distance error (front and bottom views) are reported for the reconstructions, respectively, with 336, 968, and 2165 CPs from left to right. In the horizontal axis of Figures 9(f) and 9(g), time represents the adimensional period of the cardiac cycle, where *T* = 0 indicates the time corresponding to the end-diastolic configuration.

For the reconstructions with 336, 968, and 2165 CPs, respectively, 67.6%, 74.5%, and 83% of the nodes have a distance error inferior to the pixel size, while 7.6%, 4.0%, and 2.4% of the nodes have a distance error larger than 1 mm (Figure 10(b)). Conversely, the percentage of the nodes with distance error inferior to 1/1000 mm decreases by increasing the number of CPs. This trend is due to the fact that the reconstructions with 968 and 2165 CPs could better catch the trabeculae of the LV surface resulting in a more irregular surface. Therefore, some smoothing operations were performed in these cases to preserve the mesh quality. The differences induced by the smoothing operations in the LV sac are visible also in the color maps (Figure 10(a)). By looking at them from left to right it is also noticeable how increasing the number of CPs enlarges/reduces the areas associated with a lower/higher distance error (yellow, green, cyan spots/red, and magenta spots).

As for the volume difference percentage (Figure 10(c)), the average value is 2.9%, 1.1%, and 0.9%, for the LV reconstructions with 336, 968, and 2165 CPs, respectively. This again indicates that the two latter cases provide more accurate reconstructions of the original surfaces.

#### 3.1.2. Quality

In the reconstructions with 968 and 2165 CPs some configurations (resp., 3 and 6) throughout the cardiac cycle had a few elements with a skewness higher than the threshold value of 0.9. For this reason, some manual corrections were performed with a dedicated algorithm to modify critical mesh elements in the Y-junction (which took less than to 10 minutes). Regarding the skewness distribution (Figure 10(d)) 65%, 72.7%, and 77.9% of the elements are below the threshold of 0.3 for the reconstructions with 336 CPs, 968 CPs, and 2165 CPs, respectively.

#### 3.1.3. Physiological Quantities

We also derived relevant physiological quantities from the 4D CT dataset: the LV volume (Figure 10(f)), the distance between the apex and the bifurcation center (Figure 10(g)), and the ejection fraction (EF).

The volume and the EF are reported both for the segmented geometries and the reconstructed datasets. The distance between the apex and the bifurcation center was calculated only for the reconstructed datasets. EF was 0.56, 0.58, 0.55, and 0.56, respectively, for the segmented geometry and the reconstructions with 336, 968, and 2165 CPs underlying once more the accuracy and the reliability of the method.

The distance between the apex and the bifurcation center (Figure 10(g)) follows the same trend in the three different reconstructions, with an increase in the initial configurations (the 1st configuration of the dataset corresponds to the end-diastolic LV configuration) followed by a decrease until the last configuration. This is in agreement with the evolution of LV volume until the 10th configuration, while the opposite trend after this configuration suggests that the increase in volume is not followed by a vertical elongation.

### 3.2. US and MRI Dataset

**Figure 11:**
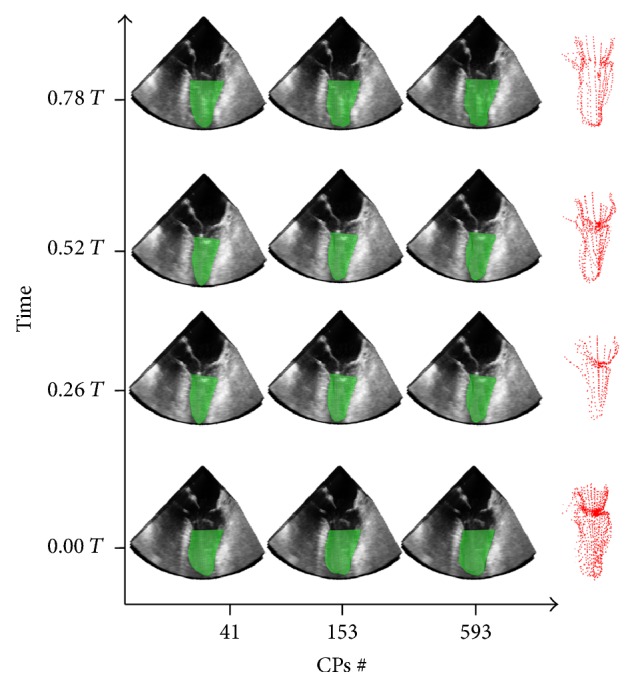
LV sac reconstructions for the 4D US dataset with 41 (left), 153 (middle), and 593 (right) at the end-diastolic configuration (1st row down), end-systolic configuration (2nd row down) CPs, and two intermediate configurations (upper rows). On the right side of the graph are the corresponding segmented point-clouds. *T* represents the adimensional period of the cardiac cycle, where *T* = 0 indicates the time corresponding to the end-diastolic configuration.

**Figure 12:**
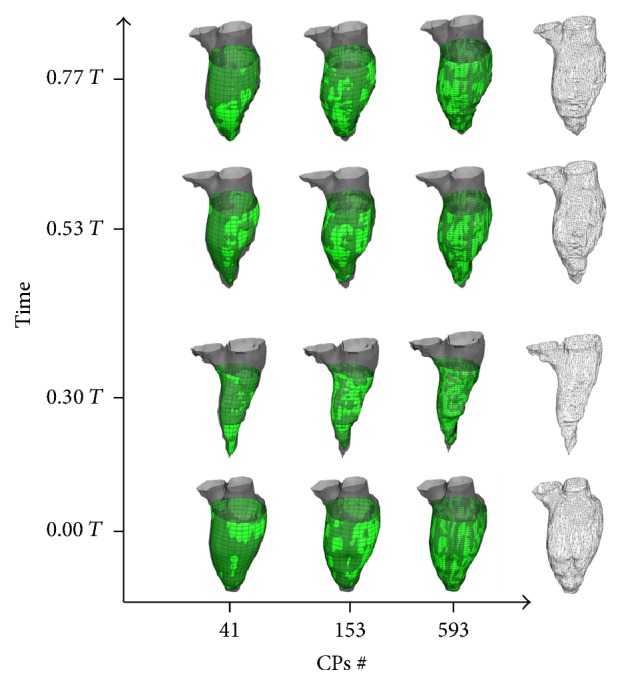
LV sac reconstructions for the 4D MRI dataset with 41 (left), 153 (middle), and 593 (right) CPs at the end-diastolic configuration (1st row row), end-systolic configuration (2nd row down), and two intermediate configurations (upper rows). On the right side of the graph are the corresponding segmented meshes. *T* represents the adimensional period of the cardiac cycle, where *T* = 0 indicates the time corresponding to the end-diastolic configuration.

In the US (Figure 11) and MRI case (Figure 12) the LV endocardial surfaces have been reconstructed up to 50% and 55% of the distance between the apex and the mitral valve centroid, respectively.

#### 3.2.1. Accuracy

**Figure 13:**
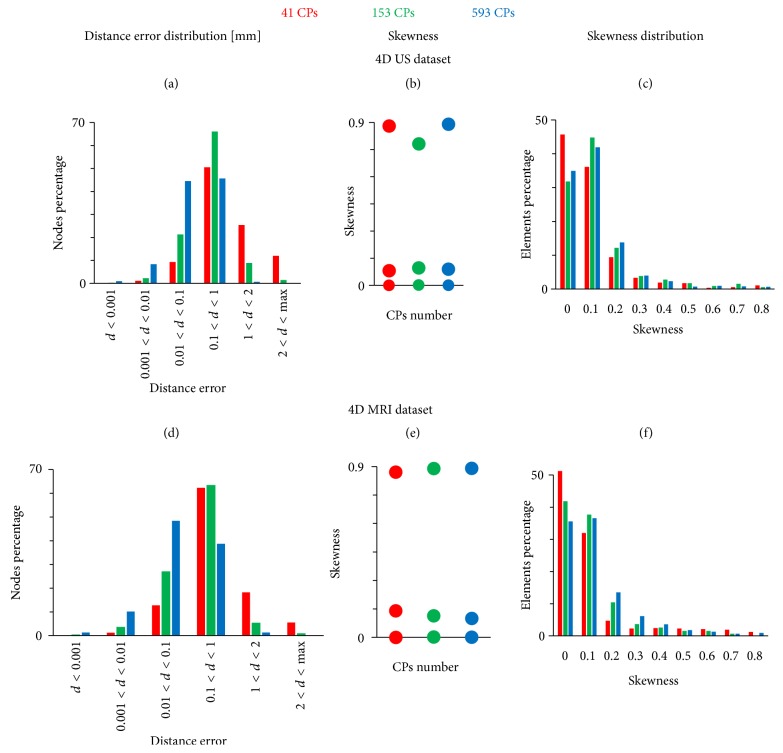
Quantities reported to evaluate the accuracy from the original 4D dataset and the quality of the newly created 4D meshes for the 4D US (a, b, c) and 4D MRI datasets (d, e, f), respectively, with 41, 153, and 593 CPs in red, green, and blue.

As expected, increasing the number of CPs from 41 to 593 resulted in a better accuracy. This leads to the following (Figures 13(a) and 13(d)):

(1) An increasing number of elements in the classes associated with a lower error, thus closer to the surface to be reproduced.

(2) For about 90% of the configurations, the maximum value of the distance error decreased.

Increasing the number of CPs, the percentage of the nodes with distance error inferior to 1 mm is 61%, 89.8%, and 99.3% and 76.4%, 94%, and 98.5%, respectively, for the US and MRI. The maximum value of the distance error decreased for almost 90% of the cases for both MRI and US.

#### 3.2.2. Quality

The skewness conditions were satisfied in every case (Figures 13(b) and 13(e)), but increasing the number of CPs did not result in an improvement of the mesh quality. Both for the US and MRI datasets the histograms of the skewness distribution (Figures 13(c) and 13(f)) highlight that around 90% of the elements had a skewness between 0.0 and 0.3.

### 3.3. Algorithm Speed

**Figure 14:**
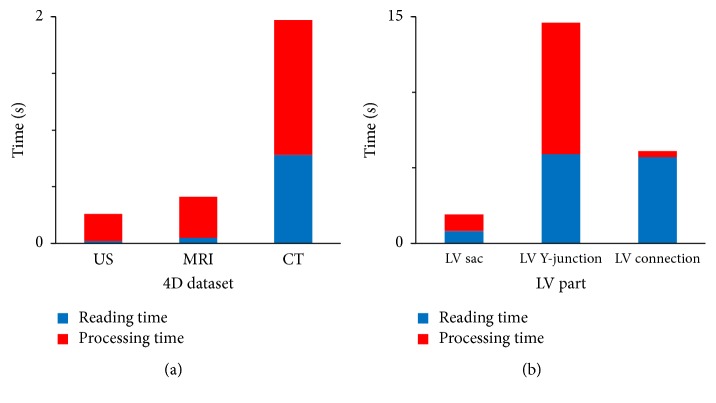
Total running time (divided into reading and processing time) is reported for the three 4D datasets (a) and for each part that form the total model in the 4D CT case (b) as index to evaluate the performance of the algorithm. The meshing involves the configuration at one instance in time.

The total running time of the algorithm is reported in (Figure 14). For the CT case the performance data refers to the reconstruction with 336 CPs, in order to have a number of CPs closer to the US and MRI case. At first glance, the total time was very different in the three cases, but this is due to the different reading time that is proportional to the dimension of the starting geometry file (resp., 0.1, 2, and 20 MB).

In the CT case, 1.97 s are required to reconstruct one LV sac, while the total reconstruction time for one complete LV geometry becomes 22.37 s. Note that 9.99 s out of these 22.37 s are related to reading the data. Most of the processing time is spent on the script that creates the high-quality structured mesh for the LV Y-junction; therefore future optimization of the algorithm should aim for this part.

## 4. Discussion

In this paper is presented a novel semiautomatic methodology to create 4D high-quality structured meshes with 1-to-1 vertex correspondence starting from 4D imaging acquisitions of the left ventricle. It has been applied to three different 4D imaging datasets (US, MRI, and CT) and all the generated 4D meshes have been reported as video file in the Supplementary Materials 1 of this paper. To the best of our knowledge, the current state of the art in 4D patient-specific cardiac geometries is mainly based on deformable models, with an extended part of the research being focused on improving the quality of the clinical images, the temporal resolution of the acquisition techniques, and the automation of the cited models.

Among the works on deformable models, among others, Lim et al. [20] reconstructed 4D patient-specific meshes of the heart with 1-to-1 vertex correspondence. In the referred case the heart was automatically meshed with triangular elements only and it took about 30 minutes to create a 4D mesh dataset of 20 frames with 20,000 nodes on their computer infrastructure. The main limitations of deformable model techniques are that they exhibit poor convergence for noisy images, large errors associated with large deformations between the neighboring frames, and the need of user-interaction to manually segment the first configuration and delineating the contours along all the configurations of the 4D dataset, which is a very laborious task also for skilled practitioner [20, 25]. The added value of our method is that it is independent of the clinical images quality, the accuracy is tunable by varying the number of control points, it is versatile both in terms of the pathophysiological state of the heart (working equally with healthy and pathological hearts) and in terms of imaging clinical modalities (CT, MRI, and US). Together with our developed code which performs a temporal interpolation of the 4D high-quality meshes, we can easily meet the temporal resolution requirements of a CFD simulation (by creating as many intermediate high-quality meshes as needed) even when starting from a 4D dataset which contains at most 30 configurations along the cardiac cycle. Furthermore, referring to the manual version of our tool (which requires the manual placement of the final CPs), it can be used in general for all the high-quality meshing problems and, in combination with tracking methods and landmarks, it could be used to include the torsion and stretching of the LV endocardium.

From a more practical point of view, the advantages related to the use of our semiautomatic morphing tool are that the operator can choose both the number of nodes (by changing the number of subdivisions of the isoparametric transformations) and the mesh element type (by defining the patch delimitated by the initial CPs); moreover, the subdivision of the LV in three different parts is useful in case that the user wants to increase the mesh density in a specific location (around the apex or the Y-junction bifurcation or in the middle band).

As quality requirements, we choose mean and maximum skewness threshold values of 0.25 and 0.9. In the cases corresponding to the 4D US, 4D MRI, and CT with 336 CPs the criteria were successfully fulfilled. For the CT data, in the Y-junction reconstructions with 968 CPs and 2165 CPs the few elements that did not meet these criteria were fixed with an additional code, which let the user manually move the CPs or the nodes in the critical spots of the mesh. For the studied case, this involved no more than 10 minutes for the corrections.

Regarding the accuracy, in the 4D CT case the percentage of nodes that has a distance error inferior to the pixel size (0.35 mm) is 67.6%, 74.5%, and 83.0%, respectively, for the 336, 968, and 2165 CPs reconstructions. These values become 92.4%, 96%, and 97.6% if we use a threshold value of 1 mm, such as what is done for the US and MRI case. In the case with 593 CPs both the 4D US and MRI high-quality meshes have a distance error inferior to 1 mm for, respectively, 99.3% and 98.5% of the nodes. Considering that the pixel size is 0.60 and 1.64, using the threshold of 1 mm can be regarded as effective. Therefore, we conclude that both the quality and the accuracy of the morphing tool have been successfully tested for each of the three 4D datasets.

For the 4D CT dataset (which reconstructs the total LV geometry), we also estimated some relevant physiological parameters, quantifying cardiac deformation and function (distance between the apex and the bifurcation center, the volume and the ejection fraction). Due to the fact that the meshes share the same connectivity, an evaluation of a distance during the cardiac cycle becomes immediately feasible with few lines of code.

The algorithm developed can build a complete high-quality LV mesh in approximately 22 s; thus a 4D dataset of 15 configurations is reconstructed in 5′30′′. In our case the computational time is affected by the number of CPs projected onto the surface but does not depend on the number of nodes of the mesh (determined by the subdivision used in each patch); therefore the subdivision of each patch is almost of free-cost resulting in a substantial saving of computational time if the user wants to change the mesh density ex post. All of the code was implemented in PyFormex, an open-source python-based software.

The main limitation of the morphing tool (in the semiautomatic implementation) is that the nodes of the reconstructed 4D meshes do not follow the material anatomical points of the LV. Therefore the resulting 4D meshes do not take into account the torsional motion of the LV, which may have an impact on the development of the intraventricular flow and may be a parameter of physiological relevance. In the cases presented, the available 4D medical image modalities (4D US, MRI, and CT) could not track the motion of the material points of the LV endocardium. However, when one can start from images with tracking and landmarks positioning (such as CMR Tissue tagging, MRI Phase contrast imaging, or Speckle-Tracking Echo), one can track the material points by means of the general technique which relies on the manual positioning of the final CPs, without using the semiautomatic algorithms for the LV sac, the atrium and the aorta, and the zone connecting both. As such, one could incorporate torsion in the meshed datasets.

Also, the mapping algorithms are not fully automatic requiring the interaction of the user, even if minimal, in the choice of (i) the two scaling factors in the LV sac application, (ii) the two picked points in the Y-junction application, and (iii) the starting border nodes for the renumbering (Step  (1) of the connection between LV sac and Y-junction). A strategy similar to the one used by Lim et al. [19] (based on greedy algorithm) could be used to automatically select the two points in the Y-junction. As future development, these steps could be fully automated.

For the CT dataset, we included the LA and aorta up to a certain length, which can be increased straightforwardly until running into principal bifurcations. On the contrary, the presence of the appendage was neglected at this stage. Future actions must be performed to accurately include the appendage and the principal bifurcations of the LA in order to segment and mesh a complete left heart model.

At this stage the morphing tool requires a 4D segmented dataset or a 4D cloud points as input to build the 4D quality meshes. Even if more and more segmentation software is going into the direction of automatic procedures, the morphing tool could be upgraded in order to be applied directly to the 4D imaging dataset. In this case the placement or projection of the CPs would be based on the grey-scale levels of the image. Also, by starting from the generated structured surface meshes the automatic development of high-quality hexahedral volume meshes will be addressed.

## 5. Conclusions

We developed a novel method capable of creating 4D high-quality meshes of the LV with 1-to-1 vertex correspondence starting from a generic 4D clinical imaging dataset. The generated 4D high-quality meshes can be used as reference to impose the LV endocardial motion in a patient-specific CFD or FSI simulation with moving boundaries and avoid convergence errors due to low quality meshes. The sensitivity of the CFD and FSI-results to choices made in morphing and meshing will be the subject of a follow-up study. The method focuses on the LV geometry but is easily adaptable to other anatomical sac or tube-like structures.

## Supplementary Materials

Supplementary Materials 4D patient-specific structured meshes of the LV endocardium from 4D clinical images.
